# Empowerment Model for The Elderly and Community to Improve the Welfare of the Elderly

**DOI:** 10.12688/f1000research.176617.2

**Published:** 2026-06-05

**Authors:** Friska Indria Nora Harahap, Mustofa Kamil, Ria Rizkia Alvi, Arief Tukiman Hendrawijaya, Bayu Pradikto, Hepy Tri Winarti, Muhammad Adil Arnady, Ahmad Syahid, Rosilawati Rosilawati

**Affiliations:** 1 Universitas Pendidikan Indonesia, Bandung, West Java, Indonesia; 2 Universitas Jember, Jember, East Java, Indonesia; 3 Universitas Mulawarman, Samarinda, East Kalimantan, Indonesia

**Keywords:** elderly empowerment; community participation; elderly welfare; community-based model; active ageing; social capital.

## Abstract

This study aims to develop and examine an empowerment model involving older people and the community to support improvements in elderly welfare. Using a mixed-methods design, the study combines quantitative assessment of welfare outcomes with qualitative exploration of empowerment processes. Quantitative data were collected from 210 elderly participants involved in community-based empowerment programs. Welfare was measured across physical, psychological, social, and economic dimensions. Data were analysed using descriptive statistics, paired-samples t-tests, and multiple regression. The results show an increase in welfare scores between pre- and post-program measurements. The mean score increased from 3.12 to 3.89 (t = 9.46, p < 0.001). These findings suggest a positive association between program participation and reported welfare improvements, although causal conclusions cannot be established due to the study design. Qualitative findings provide insights into how empowerment processes may contribute to perceived improvements in well-being.

## 1. Introduction

Population ageing is a global demographic trend that brings both opportunities and challenges. As life expectancy rises due to improvements in healthcare and living standards, societies are witnessing a growing proportion of elderly individuals. While this demographic transition is indicative of social progress, it also necessitates a reevaluation of existing elderly welfare programs, especially in developing contexts, where a shift from assistance to empowerment is required. Elderly welfare encompasses physical health, psychological well-being, social participation, and economic security. In China, for instance, as noted by Bao et al., the population aged 60 and older constitutes a significant 18.7% of the total population, highlighting the critical need for effective welfare strategies ( Bao et al., 2022). Various studies underscore the importance of promoting active ageing by creating environments that enable older people to engage in social, economic, and civic activities, thereby enhancing their quality of life ( Fung et al., 2023; Torku et al., 2021). Traditional approaches often treat older adults as passive recipients of aid, failing to foster their independence and self-efficacy ( Espinel-Jara et al., 2025; Rahmayani et al., 2024).

In many communities, welfare programs for older people tend to be fragmented, focusing predominantly on immediate assistance rather than long-term empowerment ( Huang et al., 2025; Lü & Wu, 2022). This fragmentation often results from limited collaboration among families, local institutions, and social organisations, thereby diminishing the sustainability of these initiatives ( Kung et al., 2023). Therefore, there is an urgent need for holistic community-based approaches that can effectively mobilise family support and local resources while fostering elderly empowerment. Community-based empowerment offers a promising framework for enhancing elderly welfare. This model prioritises participation, social capital, and shared responsibility within the community. Empowerment initiatives, as highlighted by Wu et al., recognise elderly individuals as valuable contributors to community life, leveraging their experiences and skills to enhance social engagement (A. K. C. Wong et al., 2022; Wu et al., 2022). The active involvement of older adults in decision-making related to welfare programs not only improves their well-being but also cultivates a sense of belonging ( Cherbuin et al., 2021).

Furthermore, research reveals a strong correlation between community engagement and life satisfaction among older adults. By involving them in meaningful activities such as volunteering and social participation, these models aim to bolster their independence and well-being ( Huang et al., 2025; Yu et al., 2021). The potential of technology, particularly Information and Communication Technology (ICT), is notably relevant, as it can facilitate social interactions and community engagement, further enhancing older adults’ quality of life ( Ivleva et al., 2024; Nakada et al., 2024).

Intergenerational practices can strengthen social cohesion and mitigate isolation among older adults ( Wu et al., 2022; Zhang et al., 2025). Studies by Serrat et al. demonstrate that fostering intergenerational interactions can markedly improve the physical and emotional well-being of elderly individuals ( Serrat et al., 2021). By bridging the generational divide, communities can benefit from the wealth of knowledge and skills of older adults while simultaneously enriching younger generations’ understanding and appreciation of ageing. Social capital plays a crucial role in empowering older adults by fostering connections and strengthening community ties. A robust sense of social capital can positively influence health outcomes and enhance participation in community activities, as observed by Ayuningtyas et al. (2022) and Ramírez-Arellano et al. (2021). Communities that actively cultivate social capital may create environments conducive to ageing in place, thereby supporting elderly individuals in maintaining independence and engagement in their local contexts ( Yu et al., 2021).

As the global population ages, there is a pressing need to innovate elderly welfare initiatives, shifting from passive assistance to active community-based empowerment. By embracing models that promote social participation, intergenerational relationships, and the harnessing of social capital, societies can create environments that support the independence and well-being of older adults. Future research and practice should focus on developing structured empowerment models that integrate elderly participation with community engagement, ensuring that their voices and contributions are valued in the discourse surrounding ageing.

Therefore, this study aims to develop and examine an empowerment model for older people and the community to improve elderly welfare. Specifically, the study seeks to analyse the model’s effectiveness in enhancing multiple dimensions of welfare and to identify key empowerment components that contribute to positive outcomes. The findings are expected to provide theoretical insights into empowerment-based ageing interventions and practical guidance for policymakers and practitioners in designing sustainable, community-driven elderly welfare programs.

## 2. Literature review

### 2.1 Concept of elderly welfare

Elderly welfare is a complex, multidimensional construct encompassing aspects critical to the well-being of older adults. This notion integrates physical health, psychological well-being, social connectivity, and economic security, recognising that these dimensions interact to shape the overall quality of life (QoL) in elderly populations ( Chatzinikolaou et al., 2021; Lin et al., 2020). Physical well-being refers to older adults’ functional capabilities, including mobility and access to appropriate healthcare services.

Research indicates that chronic conditions and physical disabilities profoundly impact older people’s ability to perform daily activities, ultimately influencing their overall quality of life ( Souza et al., 2021; Thevathasan et al., 2025). Access to healthcare facilities and effective disease management are paramount for promoting physical well-being among older adults. Health-related quality of life (HRQoL) assessments are integral in evaluating and enhancing patients’ physical capabilities by scrutinising the impacts of diseases on their functional status and overall well-being ( Noroozi et al., 2021; Owusu et al., 2025).

Psychological welfare involves factors such as emotional stability, life satisfaction, and a sense of purpose. A comprehensive assessment of well-being among older adults acknowledges the significance of mental health aspects intertwined with their psychological state ( Rohenkohl et al., 2022; Safarabadi et al., 2024). According to Schaal’s multidimensional approach, psychological well-being encompasses elements of self-acceptance, personal growth, and life meaning, which are crucial for maintaining a strong sense of identity and resilience in old age ( Alhasan et al., 2025; Weiske et al., 2020). Findings show that mental health challenges, including depression and anxiety, frequently arise among older populations and must be addressed to improve their psychological welfare ( Lazović et al., 2025; Orr et al., 2022).

Social welfare addresses the capability and opportunity for individuals to engage in meaningful social interactions and community life. Loneliness and isolation are prevalent issues that plague many elderly individuals, leading to detrimental effects on their mental and physical health ( Shahin et al., 2022; Widagdo et al., 2022). Active participation in community activities and the formation of strong interpersonal relationships are essential components of social welfare, as they significantly contribute to emotional support and a sense of belonging ( Hammad et al., 2025; Mituś et al., 2020). The interaction between social relationships and overall well-being has been well-documented, emphasising that robust social networks are vital for sustaining emotional health ( EKER et al., 2021; Tsubokawa et al., 2022).

Economic welfare encompasses income stability and the ability to meet basic needs independently. Financial security is necessary for older adults to access healthcare resources, participate in social activities, and maintain a decent standard of living ( Li et al., 2025; Seo et al., 2023). Research suggests that financial strain can exacerbate stress and negatively impact both psychological and physical health outcomes, reinforcing the interconnectedness of economic welfare with other dimensions of elderly well-being ( Antunes et al., 2025; H. Wang et al., 2021). Addressing economic vulnerabilities through social policies that promote financial independence and security can significantly enhance elderly welfare and their quality of life ( Chukkali et al., 2024; Wilder et al., 2021).

As emphasised in various studies, achieving comprehensive elderly welfare necessitates an integrated approach that harmonises healthcare interventions with social, psychological, and economic considerations ( Bartzou et al., 2024; Salarfard et al., 2025). An understanding of quality of life through a multidimensional lens affords practitioners and policymakers opportunities to design effective interventions that encompass all aspects of well-being rather than relying on singular health-focused strategies ( Giacomo et al., 2025; Gudala et al., 2025). Continuous assessment and adaptation of strategies that address the diversity of elderly individuals can further enhance their quality of life ( Chang et al., 2020; Targino, 2021). Elderly welfare is a multi-faceted construct that requires a holistic approach, interweaving physical health, psychological well-being, social integration, and economic security to enhance the quality of life for older adults.

### 2.2 Community empowerment theory

Community empowerment theory plays a crucial role in encouraging active participation and control among individuals and groups, particularly in the context of elderly welfare. This theory emphasises enabling community members to influence decisions and access resources that significantly affect their lives ( Prasetyani et al., 2023; Rahmayani et al., 2024). Empowerment is a transformative process involving capacity building, social participation, and collective action that benefits ageing populations by fostering self-sufficiency and community involvement ( Malik et al., 2020).

In essence, community empowerment strengthens social capital through enhanced trust, shared norms, and collaboration among its members ( Fafurida et al., 2024). When applied to elderly welfare, it creates an environment where older adults can engage in decision-making processes and participate actively in social activities, thereby positioning communities not just as service providers but as partners in promoting mutual support and resilience among older people, their families, and local institutions ( Bust et al., 2022). This comprehensive approach can improve the quality of life among older adults and foster a stronger sense of belonging and satisfaction within the community ( Kolesnyk et al., 2021). Integrating community empowerment into elderly welfare strategies can significantly enhance older adults’ agency, participation, and overall well-being, ensuring their voices are heard and valued in shaping their lives and communities.

### 2.3 Empowerment models for the elderly

Empowerment models for older people can be categorised into family-based, community-based, and integrated approaches, each addressing different facets of elderly welfare. Family-based models prioritise caregiving and emotional support within the household context. They enhance the quality of life for elderly individuals by ensuring that their needs are met through familial ties. However, these models often place a considerable burden on family caregivers, potentially leading to caregiver burnout ( Hidayat & Elmaghfuroh, 2024). The effectiveness of family care in enhancing elder health outcomes has been documented ( Panjaitan et al., 2024). Yet, reliance on this model may overlook the need for external support systems to alleviate the pressures families experience.

Community-based models leverage local organisations, volunteers, and social groups to promote health, social engagement, and livelihood support among older people. Programs focused on physical activity, such as community yoga or exercise initiatives, have demonstrated positive effects on both physical and mental health outcomes for older adults ( Katayama et al., 2020; Pacheco et al., 2020). Furthermore, these models improve social integration by fostering connections within the community, enhancing older adults’ overall quality of life ( Hsu et al., 2024).

Integrated models combine family support with community initiatives, creating a cohesive framework that links health services, social participation, and economic activities. Research indicates that integrated models are particularly effective in promoting independence and social engagement among older people ( Mendoza-Núñez et al., 2022). Community programs that facilitate access to healthcare and social resources tend to yield better health outcomes than single-sector interventions alone ( MacPherson et al., 2022). While family-based, community-based, and integrated empowerment models each offer valuable strategies for enhancing elderly welfare, empirical evidence suggests that integrated approaches provide the most comprehensive support for improving independence and quality of life among older adults. Despite the growing body of research on active ageing and community empowerment, existing studies tend to examine these domains in isolation or focus on specific program components rather than integrated frameworks. In particular, there remains a limited understanding of how empowerment processes translate into measurable improvements across multiple dimensions of elderly welfare. This study addresses this gap by proposing and empirically examining an integrated empowerment model that links participation, capacity building, social support, and economic engagement with multidimensional welfare outcomes. By situating empowerment as both a process and an outcome-oriented mechanism within community contexts, the study extends existing theoretical frameworks in community gerontology and active ageing.

### 2.4 Relevant previous studies

Empirical studies underscore the importance of elderly empowerment programs in enhancing self-efficacy, reducing social isolation, and fostering psychosocial well-being among older adults. Research has shown that community-based elderly empowerment initiatives can increase participation in social and economic activities, which are positively correlated with better mental health and life satisfaction ( Raharjo et al., 2024). These findings align with the premise that community involvement not only aids individual adjustment but also contributes to communal well-being.

The roles of community cadres and local leadership are critical to sustaining empowerment efforts. Studies suggest that such grassroots engagement is instrumental in driving ongoing participation and support within these programs ( Achjar et al., 2022). For example, comprehensive care initiatives that involve family and community collaboration have demonstrated significant improvements in health outcomes for older adults ( Ayuningsasi et al., 2022). However, many existing studies examine isolated program components without establishing a structured framework that effectively connects empowerment processes with measurable welfare outcomes. This gap necessitates a more integrated approach to understanding how empowerment initiatives can comprehensively address the diverse needs of the elderly population ( Putra et al., 2021). While existing literature highlights various aspects of elderly empowerment, the need for comprehensive frameworks that tie empowerment processes to successful welfare outcomes remains crucial for future research and practice.

### 2.5 Conceptual framework

Based on the conceptual framework established in this study, elderly welfare is positioned as an outcome of an integrated empowerment process that engages both older people and the community. Various empowerment components play a key role, including participation, capacity building, social support, and economic engagement. Empirical evidence suggests that community involvement mediates and strengthens the relationship between these empowerment activities and welfare outcomes, leading to improvements across physical, psychological, social, and economic dimensions of elderly life ( Attila et al., 2024; Dauphinais et al., 2021; P. Y. A. Wong et al., 2025).

Community-based interventions have demonstrated that involving older adults in social and economic activities significantly enhances mental health and life satisfaction ( Dauphinais et al., 2021; P. Y. A. Wong et al., 2025). The theme of collaborative approaches emphasises the need for local engagement, with community leaders and peer support systems shown to be vital in sustaining empowerment initiatives ( Ardhanari et al., 2021). Such local involvement allows elderly individuals to feel valued and active within their communities, which is critical for advancing their welfare and autonomy.

**Figure 1.**
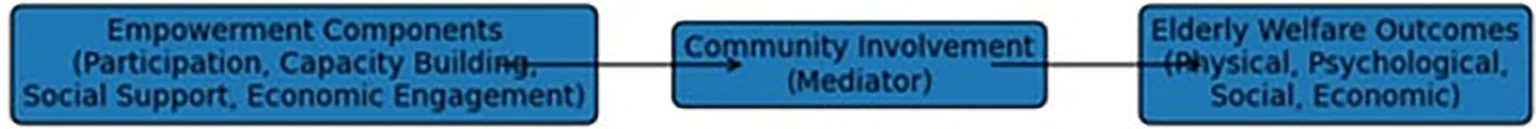
Conceptual framework of elderly and community empowerment.

However, the existing literature indicates that many studies have focused on isolated program components rather than a comprehensive framework linking empowerment processes to measurable welfare outcomes. This gap underscores the necessity for a systematic approach to integrating disparate empowerment activities into cohesive programs that yield significant welfare benefits for older people ( Elsayed et al., 2024; Mutai et al., 2020). Therefore, this study’s framework not only informs the development of research instruments but also guides data analysis and the interpretation of findings. The interplay of empowerment components within community contexts is essential for enhancing the welfare of elderly populations. The findings foster a deeper understanding of how integrated empowerment can effectively improve their quality of life. The conceptual diagram is shown in Figure 1.

Figure 1 illustrates the conceptual framework of this study, which positions elderly welfare as the outcome of an integrated empowerment process. The framework shows that empowerment components, including participation, capacity building, social support, and economic engagement, serve as the primary antecedents influencing improvements in elderly welfare across physical, psychological, social, and economic dimensions. Community involvement serves as a mediating factor, strengthening and channelling the effects of empowerment activities toward welfare outcomes. Through active engagement with families, community cadres, and local institutions, empowerment initiatives become more inclusive, sustainable, and responsive to the needs of older people. This framework underpins the development of research instruments, guides the analytical strategy, and informs the interpretation of findings in the present study.

## 3. Research methodology

### 3.1 Research design

This study adopts a sequential explanatory mixed-method design to evaluate an empowerment model aimed at enhancing elderly welfare within a community context. The initial quantitative phase will gather data on the model’s efficacy. At the same time, qualitative inquiry will follow to enrich understanding of the statistical outcomes, aligning with principles of Scientific Culture (SC) such as empirical rigour and contextual interpretation.

Mixed-methods research is particularly powerful for capturing complex phenomena, as it enables a comprehensive analysis that combines the strengths of qualitative and quantitative data ( Hicks et al., 2023; McCartney & Fu, 2023; Shaw et al., 2022). This research design has been shown to enhance methodological rigour by yielding richer, contextual insights that can elucidate and explain quantitative findings ( Eliacin et al., 2023; Nanyonga et al., 2021). Previous studies suggest that a sequential explanatory approach can yield more profound insights into participants’ lived experiences, leading to better community health outcomes ( S. Chen et al., 2023; Draper-Rodi et al., 2021). Employing this mixed-methods design not only allows for the assessment of changes in welfare outcomes but also provides a deeper understanding of how the empowerment process operates within community contexts.

### 3.2 Research setting and participants

The research was conducted in selected community settings in Medan, North Sumatra, that have successfully implemented community-based elderly empowerment programs. The participants comprised diverse stakeholders, including elderly individuals aged 60 years and above who actively engaged in empowerment activities, their family members providing daily support, community cadres and local facilitators, and community stakeholders such as regional leaders and health workers. This diverse selection aligns with best practices in community-based research, facilitating a more comprehensive understanding of the impacts of empowerment ( Djuari et al., 2023; Shi et al., 2024).

For the quantitative phase, purposive sampling was used to select 210 elderly participants who met strict criteria for program participation. This approach is known for its efficacy in identifying relevant subjects in community-based studies, thereby enhancing the relevance of the findings ( Pangastuti et al., 2023; Park et al., 2022). The qualitative phase involved 24 informants selected through maximum variation sampling, a technique that aims to capture a wide range of perspectives, allowing for a more robust qualitative analysis of empowerment experiences ( Demircan et al., 2023). Ethical principles were strictly observed throughout the study. Informed consent was obtained from all participants, confidentiality and anonymity were ensured, and participation was entirely voluntary. Special attention was given to the protection and dignity of elderly participants in accordance with ethical standards for social research. This mixed-methods approach enriches the findings with diverse participant insights, critical for understanding the complexities of elderly empowerment in community settings. Written informed consent was obtained from all participants prior to their involvement in the study.

### 3.3 Data collection techniques

Quantitative data in this study were collected using a structured questionnaire administered directly to elderly participants aged 60 years and above. The questionnaire was designed to measure key constructs related to elderly welfare and empowerment in a systematic and quantifiable manner. All items were assessed using a five-point Likert scale ranging from 1 (strongly disagree) to 5 (strongly agree), widely used in social science research to capture attitudes, perceptions, and levels of satisfaction ( Samsu et al., 2021).

The use of a structured questionnaire enables standardised data collection across respondents, facilitating statistical analysis and comparison of welfare outcomes before and after program implementation. The measured variables were derived from the conceptual framework and relevant literature, ensuring content validity and alignment with the study objectives, as in Table 1. Quantitative findings were later complemented and contextualised by qualitative data obtained through interviews ( P. Wang et al., 2023), ASs *(FGDs)* ( Yirgu et al., 2020), observations ( Khasanah et al., 2024), and document analysis to strengthen triangulation and interpretation of results ( T. Chen et al., 2024).

**Table 1.** Variables and indicators of quantitative measurement.

Variable	Dimension	Indicators	Scale
Elderly welfare	Physical welfare	Functional ability, access to health services, and physical activity	Likert 1–5
	Psychological welfare	Life satisfaction, emotional well-being, and self-confidence	Likert 1–5
	Social welfare	Social participation, peer interaction, community involvement	Likert 1–5
	Economic welfare	Income adequacy, productive activities, and financial independence	Likert 1–5
Elderly empowerment	Participation	Involvement in activities and decision-making	Likert 1–5
	Capacity building	Skills development, knowledge improvement	Likert 1–5
	Social support	Family support, community support	Likert 1–5
	Self-efficacy	Confidence in managing daily life and challenges	Likert 1–5
Community involvement	Facilitation role	Support from cadres and local institutions	Likert 1–5

### 3.4 Ethical and security considerations

This study adhered to ethical principles for social research involving human participants, with particular attention to the protection of older adults as a potentially vulnerable population. Ethical approval for this study was obtained from the Ethics Committee of Universitas Pendidikan Indonesia (Approval No. 176/UN40.F1/KEP/EC/2024) prior to data collection.

All participants received clear information regarding the study objectives, procedures, and their rights, and provided written informed consent voluntarily before participation. Confidentiality and anonymity were strictly maintained by removing personal identifiers and using coded data for analysis and reporting. Participation involved minimal risk, as the study relied on non-invasive questionnaires and interviews conducted in an age-appropriate and respectful manner. All data were securely stored in password-protected files accessible only to the research team.

### 3.5 Data analysis techniques

Qualitative data were analysed using a systematic thematic analysis approach. The coding process involved initial open coding, followed by axial coding to group categories into themes. To enhance credibility, coding was conducted by multiple researchers and discrepancies were discussed until consensus was reached. Member checking was also performed with selected participants to validate the findings. For the qualitative data, thematic analysis was employed, utilising coding, categorisation, and theme development to identify patterns related to both empowerment processes and welfare outcomes.

## 4. Results

### 4.1 Profile of elderly and community participants

Understanding the demographic and socio-economic characteristics of research participants is essential for contextualising the findings and interpreting the effectiveness of the empowerment model. Participant profiles provide insight into the living conditions, vulnerabilities, and potential resources of older adults, as well as the social environment in which empowerment initiatives are implemented. This section presents the quantitative profile of elderly respondents and the composition of qualitative informants involved in the study, as in Table 2.

**Table 2.** Profile of elderly participants.

Characteristic	Category	Frequency (n)	Percentage (%)
Age group	60–69 years	98	46.7
	70–79 years	80	38.1
	≥80 years	32	15.2
Gender	Male	87	41.4
	Female	123	58.6
Living arrangement	Living with family	152	72.4
	Living alone/with peers	58	27.6
Education level	Primary education or less	130	61.9
	Secondary education or higher	80	38.1
Income condition	Limited/irregular income	143	68.1
	Regular income	67	31.9

Table 2 shows that the majority of elderly participants were in the early elderly age group (60–69 years), indicating a relatively active population segment suitable for empowerment-based interventions. The predominance of female participants reflects broader demographic trends of higher female life expectancy. Most respondents lived with family members, suggesting that family remains a primary support system for elderly welfare, which is a critical factor in community-based empowerment models.

The relatively low educational attainment and high proportion of participants with limited or irregular income highlight the socio-economic vulnerability of the elderly population studied. These conditions underscore the relevance of empowerment programs that emphasise capacity building, social participation, and economic engagement tailored to the capabilities of older adults. In the qualitative phase, the inclusion of elderly participants, family members, and community cadres enabled triangulation of perspectives across individual, household, and community levels. This composition strengthened the analysis by capturing diverse experiences related to empowerment processes and their perceived impact on elderly welfare.

### 4.2 Description of the empowerment model

This study implemented an integrated empowerment model designed to enhance elderly welfare through active participation and community collaboration. The model is grounded in empowerment theory and emphasises the role of older adults as active agents, supported by families and community structures. Rather than focusing on a single intervention, the model integrates multiple empowerment components to address the diverse and interconnected dimensions of elderly welfare. The implementation involved coordinated roles among elderly participants, community cadres, families, and local service institutions to ensure program effectiveness and sustainability. The components and descriptions of the Elderly Empowerment Model are shown in Table 3.

**Table 3.** Components and description of the elderly empowerment model.

Empowerment component	Main activities	Key actors involved	Expected outcomes
Health empowerment	Routine health checks, physical exercise sessions, and health education	Elderly, health workers, community cadres	Improved physical health, increased health awareness
Social empowerment	Group meetings, peer-support groups, and intergenerational activities	Elderly, families, community members	Enhanced social participation, reduced social isolation
Economic empowerment	Small-scale productive activities aligned with older adults’ capacities	Elderly, community cadres, local facilitators	Increased economic engagement, improved financial independence
Psychosocial empowerment	Self-efficacy training, emotional support, life-skills development	Elderly, counsellors, community cadres	Improved psychological well-being, stronger self-confidence

Table 3 illustrates that the empowerment model is structured around four interrelated components that collectively address the physical, social, economic, and psychological dimensions of elderly welfare. Health empowerment serves as a foundational element, ensuring that elderly participants maintain functional ability and health awareness necessary for active engagement. Social empowerment activities strengthen interpersonal relationships and foster a sense of belonging, both of which are critical to preventing social isolation among older adults.

Economic empowerment activities are designed to be appropriate to the elderly capacities, emphasising participation and productivity rather than income maximisation. This component contributes not only to economic welfare but also to self-worth and independence. Psychosocial empowerment reinforces the model by enhancing self-efficacy and emotional resilience, enabling elderly participants to adapt more positively to ageing-related challenges. The active involvement of older adults in planning and implementation, supported by community cadres and families, reflects a participatory approach that strengthens ownership and sustainability. The integration of these components demonstrates that elderly welfare improvement is most effective when empowerment is addressed holistically through coordinated community action.

### 4.3 Implementation process

This section describes the implementation process of the elderly empowerment model based on qualitative observations conducted throughout the program. Observational data are essential for understanding how the model operated in practice, how participants engaged with program activities, and how contextual factors influenced implementation. The implementation process was analysed thematically through systematic coding and categorisation of observation notes, allowing patterns of participation, facilitation, and contextual variation to be identified. These findings provide practical insight into the mechanisms that supported or constrained the effectiveness of the empowerment model.

Observational findings indicate that empowerment activities were implemented regularly and consistently, with an average elderly attendance rate of 81% across program sessions. This high level of participation suggests strong acceptance of the program among elderly participants and indicates that the activities were perceived as relevant and manageable. Coding of observational data revealed frequent instances of active engagement, peer interaction, and routine participation, which were subsequently categorised under the broader theme of sustained participation.

Community cadres emerged as a central enabling factor in the implementation process. Thematic coding highlighted their roles in participant mobilisation, activity coordination, and continuity maintenance. Categories such as facilitative leadership and organisational support demonstrate that cadres acted as key intermediaries between elderly participants, families, and local service providers. Their consistent presence contributed to program stability and reinforced older adults’ motivation to participate.

Variation in implementation was also observed across community groups. Differences were most evident in the intensity of economic empowerment activities and the level of family involvement. Through thematic categorisation, communities with stronger family engagement were grouped under the theme of supportive social environment, which was associated with more consistent attendance, stronger peer support, and higher levels of collective participation among older adults. In contrast, groups with limited family involvement showed less intensive engagement, particularly in economic activities.

Overall, the thematic analysis of the implementation process highlights that while program structure and facilitation are essential, family support and community dynamics are decisive in shaping the quality and consistency of empowerment implementation. These findings help explain variations in welfare outcomes and reinforce the importance of integrating family and community support within elderly empowerment initiatives.

### 4.4 Impact on elderly welfare

This section presents the quantitative and qualitative evidence on the impact of the empowerment model on elderly welfare. Quantitative analysis was conducted to assess changes in welfare scores before and after program implementation and to identify key predictors of welfare improvement, as shown in Table 4. Qualitative findings complement and contextualise the statistical results, providing deeper insight into how empowerment processes shape the lived experiences of elderly participants.

**Table 4.** Changes in elderly welfare scores before and after program implementation.

Welfare dimension	Pre-program Mean (SD)	Post-program Mean (SD)	*t*-value	*p*-value
Physical welfare	3.18 (0.56)	3.72 (0.51)	7.84	<0.001
Psychological welfare	3.05 (0.58)	3.91 (0.47)	9.02	<0.001
Social welfare	3.10 (0.55)	4.02 (0.46)	9.88	<0.001
Economic welfare	3.14 (0.52)	3.80 (0.50)	8.41	<0.001
Overall welfare	3.12 (0.54)	3.89 (0.49)	9.46	<0.001

Table 4 demonstrates a statistically significant improvement in elderly welfare following the implementation of the empowerment model. The overall mean welfare score increased markedly indicating an increase in elderly well-being following program participation suggesting that the integrated empowerment approach is associated with improvements across multiple welfare dimensions.

**Figure 2.**
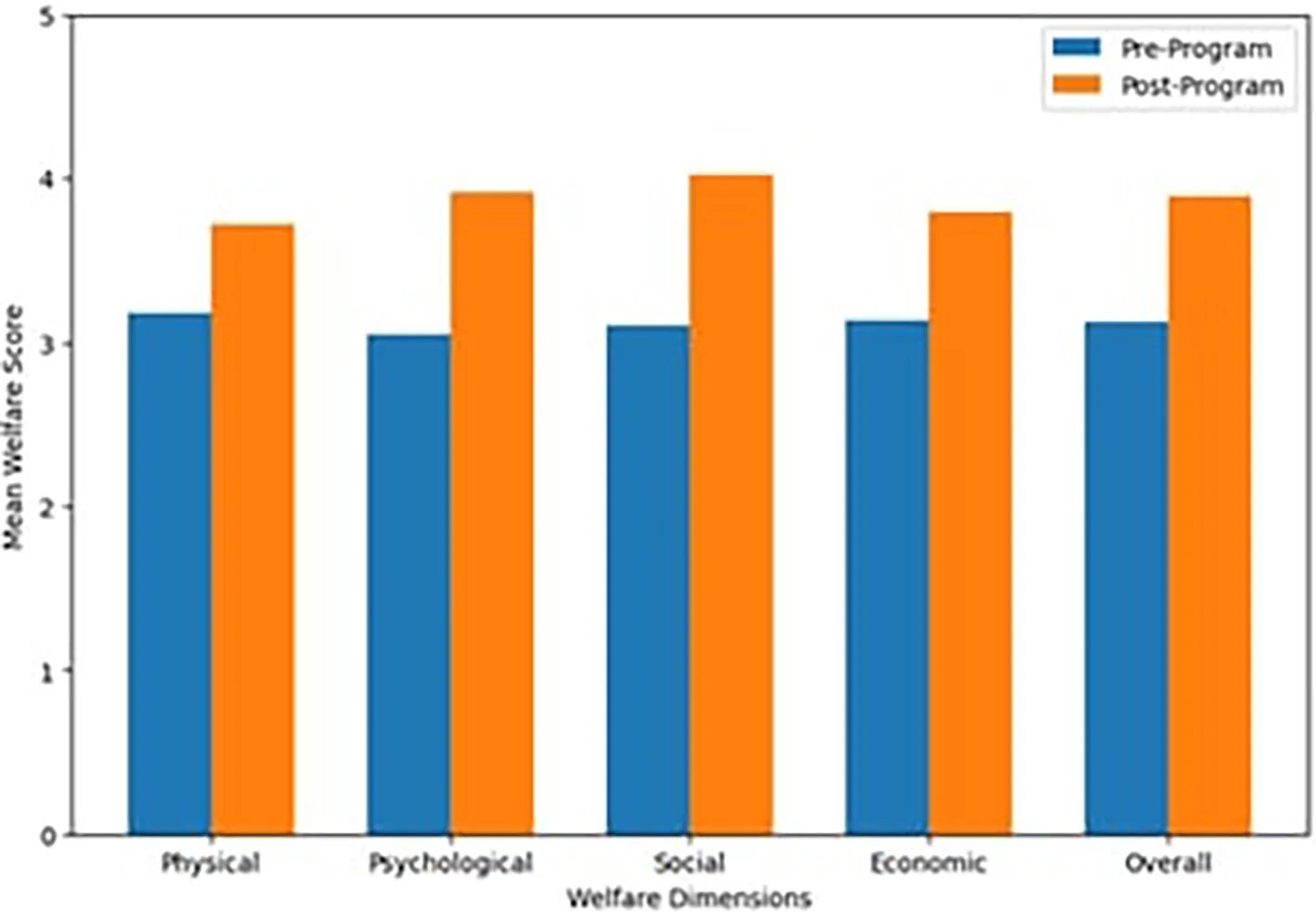
Comparison of elderly welfare scores before and after program implementation.

The most significant gains were observed in social and psychological well-being, suggesting that increased participation, peer interaction, and enhanced self-efficacy played a critical role in improving elderly quality of life. These findings are further supported by the multiple regression results, which identify community participation (β = 0.41, *p* < 0.001) and elderly self-efficacy (β = 0.36, *p* < 0.01) were significantly associated with welfare scores jointly explaining 52% of the variance in welfare outcomes (R ^2^ = 0.52). These results can be illustrated with a graph like the one in Figure 2.

Figure 2 presents a visual comparison of the mean elderly welfare scores across five dimensions—physical, psychological, social, economic, and overall welfare—before and after the implementation of the empowerment program, as reported in Table 4. The figure clearly illustrates an increase in mean scores for all dimensions following program participation.

The most notable improvements are observed in social welfare and psychological welfare, indicating that empowerment activities emphasising participation, peer interaction, and self-efficacy had a substantial impact on elderly well-being. Improvements in physical and economic welfare are also evident, though slightly less pronounced, reflecting the model’s supportive role for health-related and productive activities. Overall, the figure visually reinforces the statistical findings, demonstrating the positive and consistent effect of the integrated empowerment model on elderly welfare outcomes.

### 4.5 Qualitative findings

To complement and deepen the quantitative results, this study employed qualitative inquiry to explore how participants and community actors experienced and perceived the empowerment processes. Qualitative findings are significant in capturing changes that are not fully reflected in numerical scores, such as shifts in self-perception, social relationships, and community attitudes. Data were analysed thematically through systematic coding, categorisation, and theme development, enabling the identification of recurring patterns related to empowerment mechanisms and welfare outcomes. The qualitative results were then integrated with quantitative findings to ensure analytical convergence and interpretive rigour, in line with Scientific Culture principles.

**4.5.1 Theme 1: Increased Independence and Self-Reliance**

Elderly participants consistently described greater independence in managing daily activities and making personal decisions. This theme aligns with the quantitative improvement in psychological and overall welfare scores.

*“Before joining the activities, I depended on my children for almost everything. Now, I can manage my daily routine and even help with small tasks at home.”* (Elderly Participant, 67 years)

*“I feel more confident going out, attending meetings, and taking care of myself. I no longer feel like a burden.”* (Elderly Participant, 72 years)

**4.5.2 Theme 2: Strengthened Social Relationships and Participation**

Participants reported stronger peer relationships and a renewed sense of belonging within the community. Group activities and peer support emerged as critical mechanisms of empowerment.

“ *Meeting friends my age every week makes me happy. We share stories, exercise together, and support each other.”* (Elderly Participant, 70 years)

*“The elderly are more active now. They talk more, laugh more, and are eager to attend community activities.”* (Family Member)

This theme supports the quantitative finding that social welfare showed the most significant post-program improvement.

**4.5.3 Theme 3: Enhanced Confidence and Psychological Well-Being**

Many elderly participants expressed increased self-confidence and emotional stability, reflecting gains in self-efficacy identified in the regression analysis.

*“I feel useful again. Even small activities make me feel proud and confident.”* (Elderly Participant, 65 years)

*“They are more confident expressing opinions during meetings. This is very different from before.”* (Community Cadre)

**4.5.4 Theme 4: Shift in Community Attitudes Toward the Elderly**

Community cadres and stakeholders highlighted a significant change in how older adults are perceived—from passive beneficiaries to active contributors.

*“Now the community sees the elderly as partners. They help organise events and give advice based on their experience.”* (Community Cadre)

*“People no longer think the elderly are only recipients of aid. They are involved and respected.”* (Local Stakeholder)

The qualitative findings provide supporting evidence for the quantitative results, particularly regarding the roles of community participation and elderly self-efficacy in relation to welfare outcomes. While statistical analysis indicates measurable changes, qualitative evidence offers insight into how participants experienced these changes. Taken together, the integrated findings suggest that empowerment processes may be associated with increased independence, social engagement, confidence, and positive community recognition—factors that potentially contribute to improved elderly welfare.

## 5. Discussion

The proposed empowerment model is associated with improvements elderly welfare across physical, psychological, social, and economic dimensions. Research indicates that empowerment-oriented interventions significantly enhance overall welfare among older adults, suggesting that these changes extend beyond mere service provision. For instance, Hamedani et al. discuss the effectiveness of family-centred empowerment models in improving the quality of life for elderly patients with chronic conditions, emphasising the role of self-efficacy and community engagement ( Hamedani et al., 2021). This finding is reinforced by Emmawati et al., who highlight that therapeutic interventions such as life review therapy improve elderly well-being and foster a sense of purpose and community connection ( Emmawati et al., 2022).

Additionally, the results of community empowerment are supported by Wong et al., who find that older adults engaged in community activities exhibit better self-care behaviours, which are positively correlated with their quality of life ( A. K. C. Wong et al., 2021). This aligns with the theory of empowerment, indicating that increased control and participation can lead to improved self-esteem and well-being among elderly individuals ( J. Wang & Hoe, 2022). Programs that promote elder participation reflect their interests and capacities, leading to sustained engagement and fulfilment of welfare needs ( Fitriana, 2022).

The model’s integration of health, social, economic, and psychosocial components facilitates synergistic effects, enhancing welfare gains across these domains. This multi-faceted approach is supported by Michalski and Stępień, who argue that substantial interventions are necessary to address the challenges ageing populations face in welfare systems, emphasising the importance of holistic strategies ( Michalski & Stępień, 2021). Furthermore, the emphasis on community participation aligns with the findings of Xiang-Jing et al., who note that improved access to community health services significantly benefits older adults, particularly those with mobility challenges ( Xiang-jing et al., 2022). Comprehensive strategies that address these needs can enhance elderly well-being more effectively than traditional models focused solely on financial support ( Michalski & Stępień, 2021).

High attendance rates and consistent engagement in the empowerment program affirm that initiatives aligned with elderly interests foster acceptance and improve community welfare. Jung et al. emphasise the importance of considering the emotional and psychological impacts of community participation, advocating for structured activities that resonate with older adults to reduce feelings of loneliness and improve social integration ( Jung et al., 2023). This is crucial, as social isolation is linked to adverse health outcomes, indicating that fostering social connections through empowerment initiatives can lead to better health status and lower rates of depression among older adults ( Joanisse et al., 2025; Lee, 2021). The empowerment model successfully integrates various dimensions of welfare enhancement for older adults, showing that strong community engagement and individual self-efficacy may play an important role in improvements. The findings suggest the potential importance of adopt comprehensive, empowerment-oriented strategies in geriatric care to ensure that welfare interventions yield meaningful and lasting outcomes.

### 5.1 Comparison with previous studies

The results of the study provide compelling evidence of the positive effects of community-based empowerment on elderly well-being, particularly in enhancing social participation and psychological resilience. This aligns with the findings of Mclane et al., who argue that participation in community activities fosters health and well-being across various populations, including older adults. They note positive correlations between community engagement and improved mental health outcomes ( McLane et al., 2020). Furthermore, the relevance of community participation is echoed in research conducted by Nalla et al., which discusses the psychosocial benefits of adaptive fitness participation, reinforcing the notion that engagement in community activities enhances self-efficacy and overall quality of life ( Nalla et al., 2021).

The integration of empowerment models, as demonstrated in this study, is more effective than fragmented or sector-specific interventions. For instance, Gomes et al. elucidate the relationships among self-efficacy, physical activity, and quality of life among elderly individuals, showing that holistic approaches that incorporate multiple dimensions of empowerment lead to substantial improvements in well-being ( Gomes et al., 2023). Such findings support the argument that integrated intervention strategies are necessary for effective elder care, facilitating improvements across multiple domains simultaneously.

Moreover, this research extends existing literature by empirically demonstrating the contributions of community participation and self-efficacy to welfare outcomes through regression analysis. According to Lim et al., empowerment significantly correlates with increased community engagement; as individuals cultivate self-efficacy, they are more likely to participate actively in their communities. This pattern aligns with studies emphasising that active engagement can lead to psychological well-being and reduced feelings of loneliness among older adults ( Lim et al., 2025). The emphasis on self-efficacy as a determinant of quality of life is corroborated by findings from Azzahra et al., who indicate that enhanced self-efficacy is associated with improved emotional and physical health outcomes ( Azzahra et al., 2025).

Furthermore, the study highlights the mechanisms through which empowerment influences elderly welfare. The findings extend the assertions of Azzahra et al. that positive self-efficacy correlates with improved quality of life, indicating that those with higher self-efficacy scores experience lower levels of depression and better overall well-being, further solidifying the impact of empowerment strategies on elsupooriderly individuals ( Azzahra et al., 2025). Collectively, these studies suggest that community participation, reinforced by enhanced self-efficacy, forms a robust framework for improving elderly welfare. The combination of community engagement and self-efficacy is a powerful paradigm for enhancing the well-being of elderly populations. The research reinforces the need for integrated empowerment models that prioritise community participation, self-efficacy, and holistic welfare interventions to achieve meaningful outcomes in elderly care.

### 5.2 Implications for community development

From a community development perspective, the findings underscore the significance of positioning older adults as active social agents rather than mere beneficiaries of assistance. Empowerment models that mobilise local cadres, families, and community organisations are crucial for strengthening social capital and fostering a sense of collective responsibility. According to Attila et al., developing community capacity through collaboration among members and organisations is essential for sustainable, effective community-based interventions ( Attila et al., 2024). These collaborative approaches not only recognise the expertise within the community but also promote transparent communication and participation, creating a foundation for long-term sustainability ( Attila et al., 2024).

Community-based empowerment strategies enhance program effectiveness and embed welfare initiatives within existing community structures, ensuring their relevance and sustainability. Arizkha et al. emphasise that well-formed social networks and trust within a community facilitate participation and assistance in collective endeavours, thereby improving the quality of life and welfare of community members ( Arizkha et al., 2023). This aligns with Mendoza-Núñez et al., who advocate for empowerment models in community gerontology that recognise the potential of elderly individuals as valuable contributors to their communities ( Mendoza-Núñez et al., 2022). By promoting intergenerational solidarity and community ownership, these models support a more inclusive approach to elderly welfare.

Policymakers and practitioners should thus prioritise participatory program designs and capacity-building strategies. Huang et al. stress the importance of engaging older adults in community activities, noting that such participation can lead to greater life satisfaction and social capital ( Huang et al., 2025). This actively involves older adults in decision-making, fostering their sense of agency, which, in turn, can counter feelings of isolation and enhance their overall well-being. Additionally, community engagement has been shown to improve mental health outcomes by providing emotional support and reducing feelings of social exclusion ( Huang et al., 2025).

Furthermore, developing policies that facilitate collaborative community programs aligns with Roth et al.’s findings on leveraging community structures to solve collective problems and enhance community well-being through shared resources and capacities ( Roth et al., 2023). The results support the notion that investing in community-based structures and empowerment initiatives can significantly improve the sustainability of welfare programs. The evidence gathered points toward the importance of community empowerment in enhancing the well-being of elderly individuals. By fostering participatory approaches that engage local resources and promote collective responsibility, communities can create supportive environments that position older adults as pivotal contributors to societal welfare. This shift not only improves outcomes for older adults but also strengthens the community’s social fabric.

### 5.3 Limitations of the study

This study has several limitations that should be acknowledged, the use of a single-group pre–post design without a control group limits the ability to draw causal inferences. The observed changes in welfare scores may be influenced by alternative explanations, including natural variation, regression to the mean, Hawthorne effects, and broader social trends.

Second, all variables were measured using self-report instruments at a single point in time, which introduces the possibility of common method bias.

Third, the study does not report reliability statistics (e.g., Cronbach’s alpha), which limits the assessment of internal consistency of the measurement instruments.

Finally, limited documentation of the questionnaire and qualitative coding framework may reduce the reproducibility of the study. Future research should address these limitations by employing more rigorous designs, including control groups and longitudinal approaches.

## 6. Conclusion

This study provides evidence of positive changes in elderly welfare following participation in a community-based empowerment program. The findings highlight the potential role of community participation and self-efficacy as key factors associated with improved well-being across multiple dimensions. However, due to the limitations of the study design, these results should be interpreted as indicative rather than causal. The study contributes to the literature by offering an integrated framework that links empowerment processes with multidimensional welfare outcomes within community settings. Further research employing longitudinal and comparative designs is required to establish the effectiveness and long-term sustainability of such empowerment models, as well as to validate the relationships identified in this study across different socio-cultural contexts.

## Ethical and security considerations

This study adhered to ethical principles for social research involving human participants, with particular attention to the protection of older adults as a potentially vulnerable population. Ethical approval for this study was obtained from the Ethics Committee of Universitas Pendidikan Indonesia (Approval No. 176/UN40.F1/KEP/EC/2024) prior to data collection.

All participants received clear information regarding the study objectives, procedures, and their rights, and provided written informed consent voluntarily before participation. Confidentiality and anonymity were strictly maintained by removing personal identifiers and using coded data for analysis and reporting. Participation involved minimal risk, as the study relied on non-invasive questionnaires and interviews conducted in an age-appropriate and respectful manner. All data were securely stored in password-protected files accessible only to the research team.

## Data Availability

The datasets generated and analysed during the current study are not publicly available due to ethical and privacy considerations related to the protection of elderly participants, but are available from the corresponding author upon reasonable request ( friskaharahap@upi.edu).
