# Supplementary material for: Empowerment Model for The Elderly and Community to Improve the Welfare of the Elderly
Source: F1000Res. 2026 Jun 5;15:153. Originally published 2026 Jan 30. [Version 2] doi: 10.12688/f1000research.176617.2 (PMC13563016; doi:10.12688/f1000research.176617.2)
Supplement: Peer review history

# Reviewer report: Reviewer response for version 2

**DOI:** 10.5256/f1000research.201014.r491245

**Published:** 2026-09-14

**Authors:** Sumate Noklang

**Affiliations:** 1 Srinakharinwirot University, Bangkok, Bangkok, Thailand

I have reviewed the revised version of the manuscript. The authors have addressed the main concerns raised in my previous review. The theoretical positioning of the empowerment model has been clarified, and the descriptions of the research instruments and qualitative analytical procedures have been improved. The revised manuscript also provides a more cautious interpretation of the quantitative findings and acknowledges the limitations of the study design more appropriately.

 Overall, the revisions have satisfactorily addressed my previous concerns, and the manuscript is now of an acceptable scientific standard.

Is the work clearly and accurately presented and does it cite the current literature?

Partly

If applicable, is the statistical analysis and its interpretation appropriate?

Yes

Are all the source data underlying the results available to ensure full reproducibility?

Partly

Is the study design appropriate and is the work technically sound?

Yes

Are the conclusions drawn adequately supported by the results?

Yes

Are sufficient details of methods and analysis provided to allow replication by others?

Partly

Reviewer Expertise:

Community Psychology; Community development; Participatory Action Research (PAR); Empowerment; Social innovation; Development Education

I confirm that I have read this submission and believe that I have an appropriate level of expertise to confirm that it is of an acceptable scientific standard.

# Reviewer report: Reviewer response for version 2

**DOI:** 10.5256/f1000research.201014.r510540

**Published:** 2026-09-10

**Authors:** Ihwan Ridwan

**Affiliations:** 1 Universitas Muhammadiyah Parepare, Parepare, Indonesia

This manuscript presents a relevant and scientifically sound mixed-methods study addressing elderly and community empowerment to improve elderly welfare. The research design is appropriate, and the quantitative and qualitative approaches are suitably integrated to provide a comprehensive understanding of the empowerment process. The methods, data analysis, and interpretation are adequately presented to support the findings and conclusions. The manuscript is clearly written and provides a meaningful contribution to community-based elderly empowerment. Overall, the study is scientifically **acceptable**, and I have no major concerns regarding its methodological soundness, statistical analysis, data availability, or conclusions. I recommend the manuscript for indexing.

Is the work clearly and accurately presented and does it cite the current literature?

Yes

If applicable, is the statistical analysis and its interpretation appropriate?

Yes

Are all the source data underlying the results available to ensure full reproducibility?

Yes

Is the study design appropriate and is the work technically sound?

Yes

Are the conclusions drawn adequately supported by the results?

Yes

Are sufficient details of methods and analysis provided to allow replication by others?

Yes

Reviewer Expertise:

Nonformal Education; Community-Based Learning; Community Empowerment; Adult and Elderly Education; Community Development.

I confirm that I have read this submission and believe that I have an appropriate level of expertise to confirm that it is of an acceptable scientific standard.

# Reviewer report: Reviewer response for version 2

**DOI:** 10.5256/f1000research.201014.r510541

**Published:** 2026-09-10

**Authors:** Tri Suminar

**Affiliations:** 1 Universitas Negeri Semarang, Semarang, Central Java, Indonesia

Major revisions required

 The following issues should be addressed before the manuscript can be considered scientifically sound:

1. **Moderate all causal and effectiveness claims** arising from the single-group pre–post design.
2. **Reframe the regression analysis** as an analysis of associations rather than causal or temporal predictors.
3. **Provide full information about the measurement instruments**, including items, sources, development/adaptation procedures, validity, and reliability.
4. **Provide greater detail on the qualitative methodology**, including interview/FGD procedures, coding framework, theme development, and integration with quantitative findings.
5. **Report effect sizes and confidence intervals** for the pre–post comparisons.
6. **Provide complete regression results and diagnostics** and explain the assumptions underlying the analysis.
7. **Strengthen the theoretical contribution and novelty** of the proposed empowerment model relative to existing community gerontology and active ageing frameworks.
8. **Improve reproducibility** by providing supplementary instruments, codebook, coding framework, and anonymised data where ethically permissible.

Minor comments

 The manuscript should also be carefully proofread for language and terminology. The abbreviation “FGD” should be defined at first use. Some statements use overly strong language such as “demonstrated,” “confirmed,” or “strong positive effect,” which should be replaced with more appropriately cautious terminology. The figure presenting the pre–post results would also benefit from confidence intervals or another appropriate representation of uncertainty.

Is the work clearly and accurately presented and does it cite the current literature?

Partly

If applicable, is the statistical analysis and its interpretation appropriate?

No

Are all the source data underlying the results available to ensure full reproducibility?

Partly

Is the study design appropriate and is the work technically sound?

Partly

Are the conclusions drawn adequately supported by the results?

No

Are sufficient details of methods and analysis provided to allow replication by others?

No

Reviewer Expertise:

Traning, Nonformal Education, Community Empowerment, Adult and Elderly Education, Community Development, Educational Management, and Mixed-Methods Research

I confirm that I have read this submission and believe that I have an appropriate level of expertise to confirm that it is of an acceptable scientific standard, however I have significant reservations, as outlined above.

# Reviewer report: Reviewer response for version 1

**DOI:** 10.5256/f1000research.194694.r471209

**Published:** 2026-03-30

**Authors:** Patrick Präg

**Affiliations:** 1 CREST, ENSAE, Institut Polytechnique de Paris, Palaiseau, France

This manuscript presents a mixed-methods evaluation of a community-based empowerment model for older adults in Medan, North Sumatra. The study has a fundamental methodological limitation that substantially undermines the causal claims made, alongside several additional concerns detailed below.

 The manuscript is clearly structured and the results are presented in a broadly intelligible way. The reference list is extensive and engages with relevant literature on active ageing, community empowerment, and elderly welfare across multiple dimensions. However, the literature review remains largely descriptive rather than synthetic. There is no clear articulation of how the proposed empowerment model differs from or advances existing frameworks such as community gerontology or active ageing theory. A stronger conceptual synthesis would better justify the novelty of the contribution.

 There is an error in section 3.1, where the following sentence appears verbatim twice in succession: "Employing this mixed-methods design will not only inform the effectiveness of the empowerment model but also inform the effectiveness of the empowerment model." This should be corrected.

 The abbreviation FGD is not explained.

 The language used throughout to describe the program's effects—including phrases like "confirmed," "demonstrated," and "the program had a strong positive effect"—is more confident than the study design can support, as discussed below.

 The study uses a single-group pre–post design with no comparison condition. Welfare scores are measured before and after program implementation for the same 210 participants, and the observed improvement is interpreted as evidence that the empowerment model caused welfare gains. This inference is not warranted.

 Without a control group, there is no way to rule out a range of alternative explanations for the observed change. These include: natural recovery or seasonal variation in well-being; regression to the mean, since participants were recruited on the basis of active program involvement and may have been at a transient low point; the Hawthorne effect, whereby awareness of being studied leads to more positive self-reporting; test-retest familiarity with the questionnaire instrument; and broader secular trends in community welfare over the study period. Any or all of these could account for the pre–post difference entirely.

 The paired-samples t-test is a perfectly appropriate statistical test for detecting whether scores changed, but it cannot speak to why they changed. A randomized controlled trial, a matched comparison group, or at minimum a waitlist control would be necessary before effectiveness claims could be made. Even a non-equivalent comparison community would represent a meaningful improvement over the current design.

 Figure 2 does not have confidence intervals or comparable measures of uncertainty.

 This limitation is mentioned nowhere in the manuscript except for a brief acknowledgement that the study examined "short- to medium-term outcomes," which does not capture the problem at all. Notably, the existing peer review by Noklang also does not raise this issue.

 The mixed-methods integration itself is well-conceived, and the sequential explanatory design is appropriate for exploring how empowerment processes operate. The qualitative component adds genuine interpretive value. The academic merit of the work is therefore not in doubt as an exploratory, descriptive study—but the manuscript frames and positions itself as an effectiveness evaluation, which the design cannot support.

 Replication would not currently be possible. The questionnaire is described as measuring welfare and empowerment constructs across several dimensions, but no information is provided on how items were developed, whether the instrument was piloted, or what its psychometric properties are. Crucially, no reliability estimates (such as Cronbach's alpha) are reported for any scale, making it impossible to assess whether the observed score changes reflect genuine construct change or measurement noise.

 The qualitative analytic procedure is described only in general terms. There is no detail on how the coding scheme was developed, whether coding was conducted by more than one researcher, whether any form of inter-rater reliability was established, or whether member checking or other validation strategies were employed.

 Providing the full questionnaire, coding frameworks, and reliability statistics—ideally as supplementary materials—would substantially improve transparency and replicability.

 The use of paired-samples t-tests to assess pre–post change is appropriate for the data structure. Effect sizes are not reported, which would help contextualize the magnitude of improvement independently of sample size.

 The multiple regression analysis requires more careful interpretation. Community participation and elderly self-efficacy are described as "predictors" of welfare improvement, with R² = 0.52. However, both predictor variables and the outcome appear to have been measured at the same time point using the same self-report instrument. This constitutes a cross-sectional association, not a predictive relationship in any meaningful temporal or causal sense. The framing implies an ordering that the data structure cannot establish, and this interpretation should be substantially moderated. The possibility of common method bias inflating these associations—given that all variables are drawn from a single self-report questionnaire administered in the same setting—is also not discussed.

 The manuscript states that datasets are available upon reasonable request from the corresponding author, citing ethical and privacy constraints. This is an acceptable position for research involving human participants. However, reproducibility would be meaningfully improved by providing anonymized datasets, the full research instruments, and qualitative coding frameworks as supplementary materials. Without these, independent verification of the findings is not possible.

 The conclusions are not adequately supported, primarily because of the design issues outlined above. The conclusion that the empowerment model "is efficacious in sustainably improving elderly welfare" requires evidence of a causal effect that a single-group pre–post design cannot provide. Similarly, the claim that "community participation and elderly self-efficacy emerging as the strongest predictors of positive welfare outcomes" overstates what cross-sectional regression can establish.

 The qualitative findings are genuinely informative and the themes identified—increased independence, stronger social relationships, enhanced confidence, and shifting community attitudes—are credibly grounded in the data presented. These findings support the conclusion that participants experienced the program positively and perceived meaningful changes in their lives. This is a worthwhile contribution, but it is a different and more modest claim than the one the manuscript makes.

 The recommendations to policymakers, while sensible in spirit, should be tempered accordingly. Integrated, community-based empowerment programs may well be beneficial—but this study, as designed, does not constitute the evidence base for that conclusion.

Is the work clearly and accurately presented and does it cite the current literature?

Partly

If applicable, is the statistical analysis and its interpretation appropriate?

No

Are all the source data underlying the results available to ensure full reproducibility?

No

Is the study design appropriate and is the work technically sound?

Partly

Are the conclusions drawn adequately supported by the results?

No

Are sufficient details of methods and analysis provided to allow replication by others?

No

Reviewer Expertise:

Sociology, Gerontology

I confirm that I have read this submission and believe that I have an appropriate level of expertise to state that I do not consider it to be of an acceptable scientific standard, for reasons outlined above.

# Reviewer report: Reviewer response for version 1

**DOI:** 10.5256/f1000research.194694.r463166

**Published:** 2026-03-02

**Authors:** Sumate Noklang

**Affiliations:** 1 Srinakharinwirot University, Bangkok, Bangkok, Thailand

**Clarity of presentation and citation of current literature**

 The manuscript is clearly structured and the presentation of results is understandable, with relevant references to ageing and empowerment literature. However, the theoretical positioning of the proposed model is not sufficiently clear. The authors do not demonstrate how the model differs from or advances existing frameworks such as community gerontology or active ageing. The literature review remains largely descriptive. Stronger conceptual synthesis and clearer articulation of the model’s novelty would improve this aspect.

**Appropriateness of study design and technical soundness**

 The sequential explanatory mixed-methods design is appropriate for evaluating the model and understanding empowerment processes in community contexts. The integration of quantitative welfare outcomes with qualitative experiences of empowerment is methodologically coherent. The use of pre–post comparison with predictive analysis and thematic analysis captures the complexity of the phenomenon. Overall, the research design and procedures are appropriate and technically sound.

**Sufficiency of methodological and analytical detail for replication**

 The manuscript outlines procedures and analyses at a general level, but key details needed for replication are limited. Information on measurement instruments is insufficient, including variable structure, questionnaire items, development procedures, and validity and reliability. Qualitative analysis also lacks clarity regarding coding and theme development. Providing fuller descriptions of instruments and analytic procedures would improve transparency and replicability.

**Appropriateness of statistical analysis and interpretation**

 The use of paired-sample t-tests to assess pre–post change and multiple regression to identify predictors of welfare is appropriate. Statistical reporting and interpretation are consistent with the results and supported by qualitative evidence of increased participation and self-efficacy. The analysis and interpretation are therefore appropriate and reliable.

**Availability of underlying data for reproducibility**

 The authors state that datasets are available upon request due to ethical constraints, which is acceptable in human research. However, transparency and reproducibility would be improved by providing anonymised datasets, research instruments, or qualitative coding frameworks as supplementary materials. Such materials would enable verification and wider application of the model.

**Adequacy of conclusions supported by results**

 The conclusions are consistent with the reported significant improvements in elderly welfare and qualitative evidence of increased participation, independence, and self-efficacy. Claims remain within the scope of the data and study context without overgeneralisation. Therefore, the conclusions are adequately supported by the results.

**Minor revision is Overall assessment**

 The study provides valuable empirical evidence on community-based elderly empowerment and employs an appropriate mixed-methods research design. However, several aspects would benefit from minor clarification, particularly the articulation of the conceptual framework, the reporting of measurement validity and qualitative procedures, and the clarity of the research instruments and data used in the study.

Is the work clearly and accurately presented and does it cite the current literature?

Partly

If applicable, is the statistical analysis and its interpretation appropriate?

Yes

Are all the source data underlying the results available to ensure full reproducibility?

Partly

Is the study design appropriate and is the work technically sound?

Yes

Are the conclusions drawn adequately supported by the results?

Yes

Are sufficient details of methods and analysis provided to allow replication by others?

Partly

Reviewer Expertise:

Community Psychology; Community development; Participatory Action Research (PAR); Empowerment; Social innovation; Development Education

I confirm that I have read this submission and believe that I have an appropriate level of expertise to confirm that it is of an acceptable scientific standard, however I have significant reservations, as outlined above.
